# Treatment outcomes of multidrug-resistant tuberculosis in Hangzhou, China, 2011 to 2015

**DOI:** 10.1097/MD.0000000000021296

**Published:** 2020-07-24

**Authors:** Qingchun Li, Cynthia X. Shi, Min Lu, Limin Wu, Yifei Wu, Meng Wang, Le Wang, Gang Zhao, Li Xie, Han-Zhu Qian

**Affiliations:** a Hangzhou Center for Disease Control and Prevention, Hangzhou, Zhejiang Province, China; b Center for Interdisciplinary Research on AIDS and Department of Epidemiology of Microbial Diseases, Yale School of Public Health, New Haven, Connecticut, USA; c SJTU-Yale Joint Center for Biostatistics and Data Science, Shanghai Jiao Tong University, Shanghai, China; d Department of Biostatistics, Yale School of Public Health, New Haven, Connecticut, USA.

**Keywords:** extensively drug resistant tuberculosis, multidrug-resistant tuberculosis, retrospective cohort study, treatment outcome

## Abstract

Treatment of multidrug-resistant tuberculosis (MDR-TB) is challenging. More research is needed to understand treatment outcomes and associated factors.

A retrospective cohort study was conducted to assess trends and predictors of treatment success among 398 MDR-TB and extensively drug resistant TB patients who started treatment in 2011 to 2015 in Hangzhou, China. Sociodemographic and clinical characteristic data were obtained from the national reporting database. Chi-square test for trend was used to evaluate changes in treatment success rates over the study years, and Cox regression analysis was used to identify predictors for poor treatment outcomes.

The treatment success rate was 76% (301/398) for all participants, 77% (298/387) for MDR-TB cases and 27% (3/11) for extensively drug-resistant tuberculosis -TB cases. Treatment success increased significantly from 66% among patients who started treatment in 2011 to 85% in 2015 (*P* < .01). Of the 97 (24.4%) patients with unsuccessful treatment outcomes, 10 (2.5%) died, 64 (16.1%) failed treatment, and 23 (5.8%) were lost to follow-up. Patients who started treatment in 2013 to 2015 were less likely to have unsuccessful outcomes than those who started in 2011–2012 (adjusted odds ratio [AOR] 0.4, 95% confidence interval [CI] 0.3–0.6), patients ≥25 years were more likely to have unsuccessful outcomes than younger patients (AOR 1.6, 95% CI 1.3–2.1), and cases with kanamycin resistance was associated with three times the odds of having unsuccessful outcomes than kanamycin-susceptible cases (AOR 3.0, 95% CI 1.5–5.8).

With proper case management of MDR-TB, patients can achieve a high treatment success rate. Hangzhou's program offers clinical evidence that can be used to inform MDR-TB programs elsewhere in China and abroad.

## 1 Introduction

China has one of the world's heaviest burdens of drug-resistance tuberculosis (TB), and a better understanding is needed about the trends and treatment of multidrug-resistant tuberculosis (MDR-TB) and extensively drug-resistant TB (XDR-TB).^[1,2]^ In 2017, the World Health Organization (WHO) estimated that there were about 460,000 MDR-TB patients globally, and nearly half were in three countries: India (24%), China (13%) and Russia (10%).^[3]^ In China, 7.1% of new cases and 24% of previously treated cases were estimated to be multidrug- and rifampicin-resistant TB, respectively.^[3]^ The international community has committed to ending the TB epidemic by 2030.^[4]^ Achieving this goal in China will require earlier detection of new infections, and prompt, complete, and effective treatment of all patients diagnosed with TB, particularly drug-resistant TB.

Global studies have shown that primary resistance due to direct transmission was the main source of drug resistance among TB patients.^[5,6]^ Our 2015 study of 1326 tuberculosis patients in eastern China found a similar drug-resistance pattern.^[7]^ Effective and timely treatment renders MDR-TB patients rapidly noninfectious,^[8]^ and this emphasizes the importance of proper treatment management as the key for reining in the MDR-TB epidemic.

Of MDR patients who receive a recommended WHO treatment regimen, less than 50% achieve treatment success; this suboptimal context points to the need for newer drugs with greater efficacy and improved treatment delivery and management.^[3]^ However, there are only 3 novel drugs for treating MDR-TB in the advanced stage of development, and nine in Phase 1 or Phase 2 trials.^[9]^ Particularly since new medications are not yet available, the work of improving treatment outcomes must focus on improving diagnosis, treatment initiation, and clinical management of current drug regimens. Treatment success rates range from 34.5% to 81% globally,^[10–14]^ and factors associated with failure of treatment included higher prevalence of XDR-TB, HIV co-infection, use of standardized treatment regimens rather than individualized ones, and incomplete or non-adherent treatment.^[10,12,15–18]^ There is limited Chinese research on the outcomes of MDR-TB treatment.^[19–22]^ In this study, we aimed to evaluate the trend of treatment success across time and to identify factors associated with unsuccessful treatment outcomes in a cohort of MDR/XDR-TB patients in Hangzhou, China.

## 2 Methods

### 2.1 Study setting, design, participants and database

Hangzhou is the capital city of Zhejiang Province in eastern China. It comprises 13 districts, one county-level city and two counties, and has 7.2 million local residents and over 2 million migrant population. There are thirteen hospitals that have TB care services; of these, there are two hospitals with the capacity to treat drug-resistant TB. We constructed this retrospective cohort study using de-identified information extracted from the Tuberculosis Information Management System (TBIMS), a national online database maintained by Chinese Center for Disease Control and Prevention (China).^[23]^ Because TB is a notifiable disease in China, all diagnosed cases are reported to TBIMS, and this database serves as a central repository for TB diagnosis, treatment, and monitoring data. MDR-TB is defined as resistance to the two most common anti-TB drugs isoniazid (INH) and rifampin (RIF), and XDR-TB is defined as resistance to INH and RIF plus any fluoroquinolone and at least one of the three second-line injectable drugs (amikacin, capreomycin, and kanamycin).^[3]^ This study includes nearly all diagnosed MDR-TB and XDR-TB patients who started treatment from 2011–2015 in Hangzhou. Patients were excluded from the study if they: were diagnosed with MDR-TB or XDR-TB but did not start treatment during the study period; were newly diagnosed; or did not have a documented treatment outcome. Variables abstracted from TBIMS were socio-demographic (gender, age, occupation, residence, ethnicity) and clinical information (eg, year of starting treatment, TB treatment history, results of sputum microscopy, culture and drug sensitivity testing [DST], duration of therapy, treatment outcome). Paper records were reviewed if a case's electronic TBIMS record had missing data or logical errors. Personal identifiers were removed before data was used for analysis; no consent was obtained from individual TB patients. The study was approved by the ethics committee of Hangzhou City Center for Disease Control and Prevention.

### 2.2 TB diagnosis, treatment and management

Study participants were diagnosed and treated per the standard-of-care guidelines in Hangzhou, which were revised in 2010 and implemented with support from the Global Fund to Fight AIDS, Tuberculosis, and Malaria (Global Fund).^[7]^ Sputum samples were collected before treatment initiation and examined through acid-fast bacilli smear microscopy and culture. DST was performed for all smear-positive TB patients and for all high-risk individuals, including patients with recurrent TB; close contacts of MDR-TB patients; patients who experienced treatment failure, relapse, or retreatment; and patients who remained smear-positive at the end of the second or third month of the initial treatment. Conventional biochemical tests for drug sensitivity test were performed using the proportion method on L-enstein-Jensen medium, with the following concentrations: 0.2 micrograms per milliliter (μ/mL) for INH, 40 μ/mL for RIF, 2.0 μ/mL for ethambutol, 2.0 μ/mL for ofloxacin, 4.0 μ/mL for streptomycin, and 30 μ/ml for kanamycin. The critical growth proportion for drug resistance was set at 1%. Additional details on TB diagnosis and DST are described elsewhere.^[7]^

All bacteriologically confirmed MDR-TB and XDR-TB patients were referred to MDR-TB-designated hospitals. Treatment regimens (standardized or individualized) were decided upon by a MDR-TB physician panel based on the patients’ DST results and history of previous TB treatment according to the National Guidelines of Chemotherapy of Drug-Resistant Tuberculosis.^[24]^ Regimens included at least five drugs, including an injectable agent and fluoroquinolone, plus 2 to 3 second-line drugs (eg, cycloserine, protionamid, aminosalicylate, ethionamide, thioacetazone) and any susceptible first-line drugs (eg, pyrazinamide, ethambutol, rifapentine, rifabutin). The duration of treatments was at least 24 months, including 6 to 12 months of the injection phase and 18 to 24 months of the continuous phase. According to the Directly Observed Treatment, short-course (DOTS) Plus guidelines, the patients received DOTS and monitoring for side effects by supervisors, who were doctors from local health care centers, hospital nurses, and trained family members. Patients attended monthly follow-up clinical examinations for sputum microscopy, culture, and prescription refills.

### 2.3 Treatment outcome definitions

According to the national guidelines,^[24]^ treatment outcomes were assessed by physician panels in the MDR-TB-designated hospitals primarily based on the follow-up sputum microscopy, culture, and clinical monitoring. Study participant treatment outcomes were categorized as successful (cure or treatment completion) or unsuccessful (treatment failure, loss to follow-up, or death). Treatment outcomes were defined as follows: cure was defined as treatment completion and having ≥ 5 consecutive negative sputum cultures during the last 12 months of treatment or having ≥ 3 consecutive negative cultures following a positive culture. Treatment completion was defined as finishing the treatment regimen without evidence of failure but with inadequate bacteriological records to be defined as a cure, for example, <5 bacteriological exams during the last 12 months of treatment. Treatment failure was defined as ≥2 positive sputum cultures among the final five cultures, or one positive culture of the final three cultures during the last 12 months of treatment. Death was defined as all-cause mortality. Loss to follow-up was defined as having missed medical appointments for more than two consecutive months. Treatment outcome was defined a dichotomous variable: successful if patients were cured or completed treatment during the study years, and otherwise, unsuccessful. For all study participants who started treatment during 2011 to 2015, their treatment outcomes were assessed as of December 31, 2015.

### 2.4 Statistical analysis

In this retrospective cohort study, follow-up time was defined as the time from the date of initiating MDR-TB treatment to the date of finishing treatment. Time of censoring was defined as the time from the date of initiating MDR-TB treatment to the date of the last follow-up or December 31, 2015. Data on individuals known to have died or been lost to follow-up were censored at the date of death (if known) or the date of their last visit. Frequencies and proportions were used to summarize categorical variables, and medians were used to summarize continuous variables. Fisher's exact test and Chi-square test for trend was performed to compare differences in treatment success rates over 5 calendar years. Cox regression model was fitted to identify predictors for unsuccessful treatment. Statistical significance was defined as a two-sided *P* value < .05. All statistical analyses were conducted using SPSS version 19.0.

## 3 Results

### 3.1 Demographic and clinical characteristics of participants

From 2011 to 2015, a total of 25,081 TB cases were diagnosed in Hangzhou, and 8,806 (35.1%) were sputum smear-positive. Of these positives, 537 (6.1%) were diagnosed as MDR/XDR TB cases. About three-quarters (N = 404) of MDR/XDR TB cases started treatment. After excluding 5 patients who were still on treatment at the time of data extraction and one who had missing information, we included 398 patients in this analysis (Table 1).

**Table 1:**
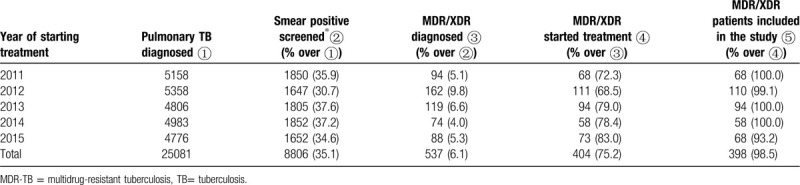
Cascade of diagnosis and treatment of multidrug-resistant tuberculosis in Hangzhou, China, 2011–2015.

Prior to treatment initiation, all participants were culture-positive, and 388 (97.5%) were smear-positive. Of the 398 participants, 387 (97.2%) had MDR-TB and 11 (2.8%) had XDR-TB. Table 2 summarizes the demographic and drug resistance characteristics: 71.4% were male, 76.4% were aged between 25–64, and 74.1% were farmers or migrant workers. The vast majority (92.7%) of participants had a history of TB treatment. The prevalence of resistance to individual TB drugs was: streptomycin 62.8%, ethambutol 44.2%, ofloxacin 10.3%, and kanamycin 5% (Table 2).

**Table 2:**
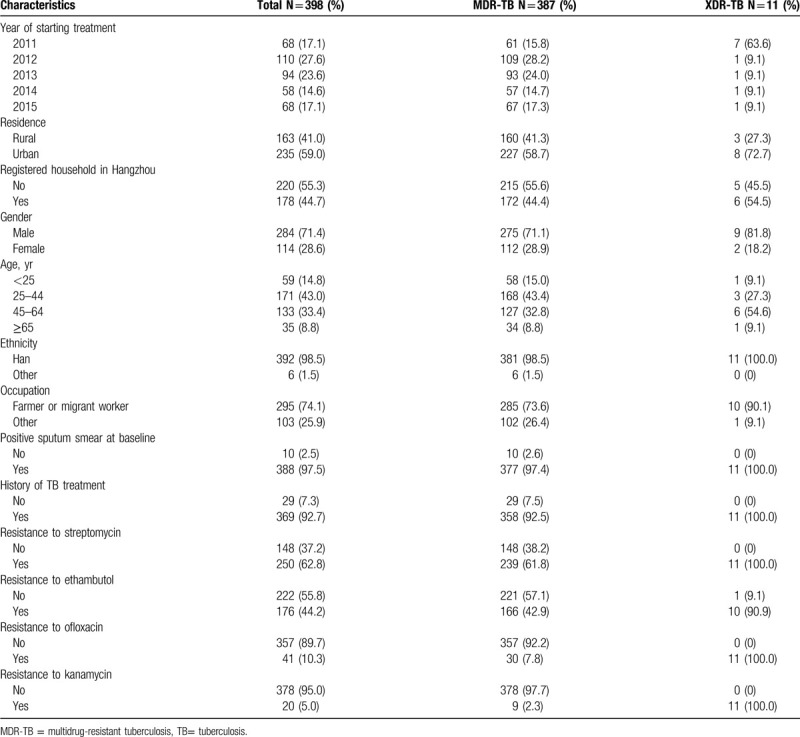
Characteristics of 398 MDR-TB patients in Hangzhou, China.

### 3.2 Treatment outcomes

In this study, the overall treatment success proportion was 75.6% (N = 301) comprising 284 patients (71.4%) deemed cured and a further 17 (4.3%) who achieved treatment completion. Of all 398 patients, the mean duration of treatment was 22.5?.3 months. The percentage of cases achieving culture conversion was 95.7% (N = 381), and the mean time to culture conversion was 84.5 days (standard deviation [SD] 56.6). Of the 388 cases who were smear-positive at baseline, 378 (97.4%) achieved sputum smear conversion with a mean time to sputum smear conversion of 51.6 days (SD 42.1). Treatment success was significantly higher for 77.0% (N = 298/387) for MDR-TB than for 27.3% (N = 3/11) for XDR-TB participants (*P* < .01). Of 97 (24.4%) unsuccessful treatment cases, 10 (2.5%) died, 64 (16.1%) failed treatment, and 23 (5.8%) were lost to follow-up (Table 3). A significant positive trend for treatment success was observed among participants who started treatment from 66.2% in 2011 to 85.3% in 2015 (*P* < .01).

**Table 3:**
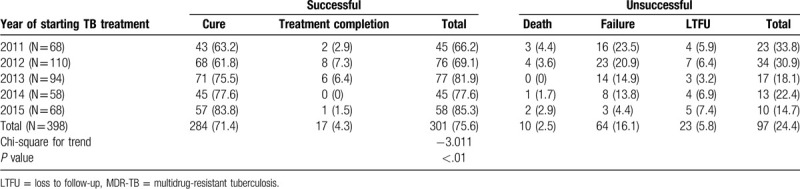
Treatment outcomes of MDR-TB patients in Hangzhou, China, 2011–2015.

### 3.3 Predictors for unsuccessful treatment outcomes

Univariate analysis identified five factors that were significantly associated with an unsuccessful treatment outcome, including year of starting treatment, residence, age, occupation, and resistance to kanamycin. These variables were included in the multivariate analysis, and 3 factors were found to be independently associated with an unsuccessful treatment outcome: year of starting treatment in 2013 to 2015 (compared to years 2011–2012; adjusted odds ratio [AOR], 0.4; 95% confidence interval [CI] 0.3–0.6; *P* < .01), older age (≥25 years vs < 25 years; AOR 1.6; 95% CI 1.3–2.1; *P* < .01), and resistance to kanamycin (AOR 3.0; 95% CI 1.5–5.8; *P* < .01). There was no statistical difference between migrants and local household registered residents (Table 4).

**Table 4:**
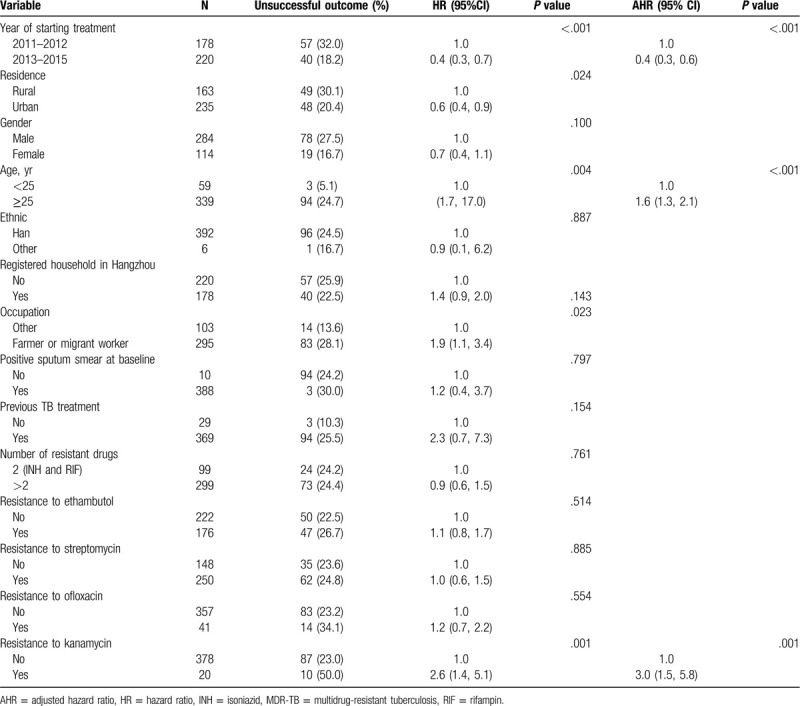
Predictors of an unsuccessful treatment outcome (treatment failure, loss to follow-up or death) among 398 MDR-TB patients in Hangzhou, China, 2011–2015.

## 4 Discussion

The average success rate of MDR-TB treatment in Hangzhou was 75.6% among patients who started treatment during 2011 to 2015, peaking at 85% among patients started treatment in 2015. To our knowledge, this was the highest treatment success rate of MDR-TB patients to be reported in mainland China.^[19–22,25,26]^ This marks Hangzhou as the first Chinese city to achieve the WHO target of 75% treatment success.^[27]^ Treatment success rates reported in other Chinese cities were all under 70%,^[19–21,25,26,28,29]^ and the cure rate ranged from 50% to 60%.^[30,31]^ Our study reaffirms a previous study that showed Hangzhou as having a higher MDR-TB treatment success rate (73.7%) than other areas of China.^[22]^ This study's success rate is higher than rates seen in most resource-limited countries^[15,32–34]^ but lower than success rates of resource-rich counties.^[11,35,36]^ When compared to cure rates from other countries, we should note that the successful treatment rate in our study included both cure and completion of treatment according to Chinese guidelines; the successful treatment rate (75.6%) was slightly higher than the cure rate (71.4%).

This is the first study in China to report an increasing trend in successfully treating MDR-TB, which we observed across five years. This temporal improvement in treatment outcomes mirror a decline of MDR-TB prevalence during the same time period in Hangzhou.^[7]^ Improving treatment success and decreasing prevalence of MDR-TB in Hangzhou points to the need to prioritize effective treatment and management of MDR-TB cases in TB prevention and control programs. Studies have shown that MDR-TB is more likely to result from transmission rather than acquisition, and improved diagnosis and treatment of MDR-TB patients will reduce the spread of MDR-TB.^[5,6]^

Our study identified numerous predictors for an unsuccessful MDR-TB treatment outcome. Older age was associated with a poorer outcome, which is consistent with the literature.^[21,37,38]^ A British study showed that the mortality risk of MDR-TB patients can almost double for every 10-year increase in age,^[37]^ and another study showed that older patients have a higher incidence of co-morbidities that can worsen disease progression and increase the risk of poor outcomes.^[20]^ Kanamycin-resistant patients were also more likely to have an unsuccessful treatment outcome. Previous research has found that susceptibility to kanamycin quadrupled the likelihood of culture conversion,^[10]^ which is a prognostic marker of successful treatment;^[39]^ thus, resistance to kanamycin may decrease the chance of culture conversion. Patients who started treatment during 2011–2012 were more likely to have an unsuccessful treatment outcome compared to those who started in 2013–2015, and we attribute this to improved implementation of the of MDR-TB treatment program in later years under the DOTS-Plus program—a DOTS program with components for MDR-TB diagnosis, management, and treatment.

Unsurprisingly, we found that XDR-TB cases had markedly worse outcomes compared to MDR-TB. Only 3 of 11 XDR-TB cases (27%) achieved a successful treatment outcome, which is consistent with studies in elsewhere in China reporting 9- 30% success.^[19,40,41]^ We had a small number of XDR-TB cases (N = 11), comprising only 2.8% of the study population. In contrast, laboratory surveillance of Zhejiang Province— which includes Hangzhou—places the XDR-TB prevalence at 6.4% of MDR-TB cases.^[2]^ No recent representative national estimates are available, but large-scale reports in China have found a prevalence of 75% to 8% of XDR-TB among MDR-TB cases; ^[42,43]^ globally, this figure is estimated to be 8.5%.^[3]^ A more comprehensive investigation of XDR-TB treatment is warranted. Our treatment results are a reminder that while XDR affects only a small proportion of MDR-TB cases, uninterrupted XDR-TB transmission poses dire consequences for public health and clinical outcomes.^[44]^

China's healthcare infrastructure and trained workforce capacity are not on par with those of fully developed nations, and in this resource-limited context, it is a laudable achievement for Hangzhou's TB control program to have met the WHO's target for MDR-TB treatment success. We suggest several reasons for the high successful treatment rate in Hangzhou from 2011 to 2015. First, the vast majority of MDR-TB cases were treated at the Zhejiang Province Center for TB Diagnosis and Treatment, which is located in the Hangzhou Red Cross Hospital. A MDR-TB physician panel developed individualized treatment plans for MDR-TB patients, which is likely to reduce misdiagnosis and mistreatment risks. Second, Hangzhou Center for Disease Control and Prevention had a designated staff member in charge of follow-up for MDR-TB patients and coordinating treatments between the Hangzhou Red Cross Hospital and Community Health Centers, and physicians in Community Health Centers established 1-to-1 treatment relationships with patients. Third, studies have shown that treatment cost is a strong predictor for treatment adherence and outcome of MDR-TB cases.^[45]^ The Global Fund in China invested heavily in scaling-up access to diagnosis and treatment of MDR-TB during the study years,^[43,46]^ and this program covered all costs of MDR-TB treatment, including patient transportation, which had a significant impact on initiation and adherence. Notably, migrant workers in China typically face challenges in accessing healthcare, but in our study, there was no statistical difference in treatment success between migrants and local residents due to the equitable financial coverage from the Global Fund. Hangzhou's experience offers valuable and timely guidance on how to strengthen MDR-TB control in China. By studying, adapting, and implementing Hangzhou's TB treatment guidelines and practices, other Chinese cities could improve the treatment outcomes of MDR-TB patients. However, while we are optimistic about the future of MDR-TB control in Hangzhou, we also urge caution in interpreting and generalizing these results. China has transitioned from being a Global Fund recipient to a Global Fund donor in the past five years, and as of 2018, only 2% of TB financing was drawn from international sources,^[3]^ which means that the financing for TB programs relies on the Chinese government. In order to continue the progress made under the China-Global Fund partnership, China must commit to sustained funding for TB prevention and control programs, particularly MDR-TB.^[47,48]^

Because this was a retrospective study design, we faced some limitations in data availability. First, some important variables such as adverse reactions, body mass index, specific treatment regimens, migration, comorbidities, and HIV co-infection, which had been reported to be predictors of poor treatment outcomes elsewhere,^[10,12,28,49]^ were not available for inclusion in this study. Second, not all monthly sputum samples were obtained from each patient, and the missing information may be a source of misclassification bias.

This study showed that Hangzhou has achieved high success in achieving treatment success for MDR-TB and has met the WHO treatment target. Treatment success rates increased from 2011 to 2015, which suggests improved implementation of MDR-TB control programs. With proper treatment and management of MDR-TB and XDR-TB, other Chinese programs could also improve treatment outcomes by adopting practices shown to be effective in Hangzhou.

## Author contributions

**Conceptualization:** Qingchun Li, Han-Zhu Qian

**Formal analysis:** Qingchun Li, Cynthia X. Shi

**Funding acquisition:** Qingchun Li

**Investigation:** Qingchun Li, Min Lu, Limin Wu, Meng Wang, Le Wang

**Laboratory tests:** Yifei Wu

**Project administration:** Li Xie

**Supervision:** Gang Zhao, Han-Zhu Qian

**Writing – original draft:** Qingchun Li

**Writing – review & editing:** Cynthia X. Shi, Han-Zhu Qian.
